# Age Heaping and Accuracy of Age Data Collected During a Community Survey in the Yavatmal District, Maharashtra

**DOI:** 10.4103/0970-0218.69256

**Published:** 2010-07

**Authors:** Geeta S Pardeshi

**Affiliations:** Department of Preventive and Social Medicine, Dr. Shankarrao Chavan Government Medical College, Nanded, Maharashtra, India

**Keywords:** Age accuracy index, age heaping, Myer’s blended index, Whipple’s index

## Abstract

**Background:**

Age is an important variable in epidemiological studies and an invariable part of community-based study reports.

**Aims:**

The aim was to assess the accuracy of age data collected during community surveys.

**Settings and Design:**

A cross-sectional study was designed in rural areas of the Yavatmal district.

**Materials and Methods:**

Age data were collected by a house-to-house survey in six villages. An open-ended questionnaire was used for data collection.

**Statistical Analysis:**

Age heaping and digit preference were measured by calculating Whipple’s index and Myers’ blended index. Age Ratio Scores (ARS) and Age Accuracy Index (AAI) were also calculated.

**Results:**

Whipple’s index for the 10-year age range, i.e., those reporting age with terminal digit “0” was 386.71. Whipple’s index for the 5-year range, i.e., those reporting age with terminal digit ‘0’ or ‘5’ was 382.74. Myer’s blended index calculated for the study population was 41.99. AAI for the population studied was 14.71 with large differences between frequencies of males and females at certain ages.

**Conclusion:**

The age data collected in the survey were of very poor quality. There was age heaping at ages with terminal digits ‘0’ and ‘5’, indicating a preference in reporting such ages and 42% of the population reported ages with an incorrect final digit. Innovative methods in data collection along with measuring and minimizing errors using statistical techniques should be used to ensure the accuracy of age data which can be checked using various indices.

## Introduction

Age is an important study variable in demography and epidemiological studies. It is a socio-demographic variable related to the host in descriptive studies and also a commonly assessed risk factor in analytical studies. The accuracy of age data collected by house-tohouse surveys varies in different set-ups and depends on numerous factors. This is clearly indicated in studies which describe the age-related data of census from different countries.(1,2)

Different set-ups have different social values attached to age. A variety of irregularities and misstatements have been noted with respect to age-related data.(3) Misstatement of age is a common example of content error in census and surveys. Of these irregularities, age heaping is a common phenomenon. Age data frequently display excess frequencies at round or attractive ages, such as even numbers and multiples of 5 leading to age heaping. Age heaping is considered to be a measure of data quality and consistency.

This study describes age heaping and assesses the accuracy of age data collected during a community survey in the Yavatmal district of Maharashtra state.

## Materials and Methods

### Data collection

The age data were collected during a survey in six villages of the Yavatmal district in Maharashtra state in September 2006. The six villages were selected by simple random sampling using the lottery method. The final year students of a college of social work were selected and trained for data collection. A pretested questionnaire was used for data collection. A house-to-house survey was conducted in the selected villages.

The age data were collected as per the information given orally by the respondents present in the house during the survey. The age of the persons not available during the survey was obtained from their family members who acted as proxy informants. No records, birth registers, or birth certificates were crosschecked to confirm the age data.

A meeting with the interviewers was conducted to note their experiences during data collection. The investigator supervised data collection to observe the process of reporting age.

### Measurement of age heaping and age accuracy

Age heaping and digit preference were measured by calculating Whipple’s index and Myers’ blended index. Age Ratio Scores (ARS) and Age Accuracy Index (AAI) were also calculated.

Whipple’s index detects a preference for ages ending in 0, 5, or both. Whipple’s index is constructed for the age group of 23–62 years using the following formula:

$$\mathrm{Whipple}’s\;\mathrm{index}\;\mathrm{for}\;\mathrm{the}\;5-year\;\mathrm{range}\;=\;\frac{\sum \left(P_{25}\;+\;P_{30}\;+\;P_{35}\;+\ldots +P_{60}\right)\;\times \;100}{1/5\;\sum \left(P_{23}\;+\;P_{24}\;+\;P_{25}\;+\ldots +P_{62}\right)}$$

$$\mathrm{Whipple}’s\;\mathrm{index}\;\mathrm{for}\;\mathrm{the}\;10-year\;\mathrm{range}\;=\;\frac{\sum \left(P_{30}\;+\;P_{40}\;+\;P_{35}\;+\ldots +P_{60}\right)\;\times \;100}{1/10\;\sum \left(P_{23}\;+\;P_{24}\;+\;P_{25}\;+\ldots +P_{62}\right)}$$

Whipple’s index varies from 0 to 500. A value of 0 indicates that digits ‘0’ and ‘5’ are not reported, 100 means there is no preference for ‘0’ or ‘5’, and a maximum of 500 is seen when only the digits ‘0’ and ‘5’ are reported in the age data. The inference about age distribution based on this index is as follows: <105 = highly accurate; 105–109.9 = fairly accurate; 110–124.9 = approximate; 125–174.9 = rough; ≥175 = very rough.

Myer’s blended index is calculated for the age above 10 years and shows the excess or deficit of people in ages ending in any of the 10 digits expressed as percentages. It is based on the assumption that the population is equally distributed among the different ages. The steps in the calculation of Myers’ blended index are as follows:

1. Sum of populations ending in each digit over the whole range starting with the lower limit of the range (e.g., 10, 20, 30, 40,….; 11, 21, 31,….)
2. Ascertain sum excluding the first population combined in step 1 (e.g., 20, 30, 40,….; 21, 31, 41,….)
3. Weight the sums in steps 1 and 2 and add the results to obtain a blended population (e.g., weights 1 and 9 for 0 digit, weights 2 and 8 for 1, etc.)
4. Convert distribution in step 3 into percentages.
5. Take the deviation of each percentage in step 4 from 10.0, which is the expected value for each percentage.
6. A summary index of preference for all terminal digits is derived as one half of the sum of the deviations from 10.0%, each without regard to signs.

ARS are calculated for age up to 74 years and are defined here as the ratio of the population in a given age group to one-third the sum of the population in that age group and in the preceding and following groups, multiplied by 100. The age ratio is expressed for a 5-year age group as follows:

ARS for Pa5 = Pa × 10051/3 Pa-5 + Pa55 + Pa+55

where _5_P_a_ is the population in the given age group, _5_P_a-5_ is the population in the preceding age group, and _5_P_a+5_is the population in the following age group.

In the absence of extreme fluctuations in the past vital events, the age ratios for all age groups should be about equal to 100. The sum of the deviations from 100 of the age ratios for males divided by number of age groups gives the mean deviation for males and the same procedure also gives the mean deviation for females. The average of the mean deviations of males and females is a measure of the overall accuracy of the age data, i.e., age accuracy index.

## Results

The age data of a total of 4304 people in 823 households were collected during the survey.

The total population in the age group ‘23–62’ was 2017. Among them, the population reporting age ending in ‘0’ was 780 and those reporting age with the terminal digit of ‘5’ were 764. Thus Whipple’s index for the 10-year age range, i.e., for those reporting age with terminal digit ‘0’, was 386.71. Whipple’s index for the 5-year range, i.e., for those reporting age with terminal digit ‘0’ or ‘5’, was 382.74.

The total population in the age group ‘above 10 years’ was 3571. Table 1 describes the steps in the calculation of Myers blended index. The Myers blended index calculated for the study population was 41.99.

**Table 1:** Calculation of preferences indices for terminal digits by Myers’ blended method

Terminal digit, *a*	Population with terminal digit		Weight for		Blended population		
	Starting at 10+*a*	Starting at 20+*a*	Column 1	Column 2	Number (1*3+2*4)	Percent distribution	Deviation of percentage from 10 (6-10)
	1	2	3	4	5	6	7
1	138	78	2	8	900	2.8776	-7.12
2	312	193	3	7	2287	7.31	-2.68
3	187	86	4	6	1264	4.04	-5.95
4	152	54	5	5	1030	3.29	-6.70
5	952	859	6	4	9148	29.24	19.24
6	196	85	7	3	1627	5.20	-4.79
7	156	74	8	2	1396	4.46	-5.53
8	256	131	9	1	2435	7.784	-2.21
9	94	28	10	0	940	3.00	-6.99
Total (irrespective of sign)	3571	2602			31281	100	83.99
Summary index of age preference							41.99

**Figure 1:**
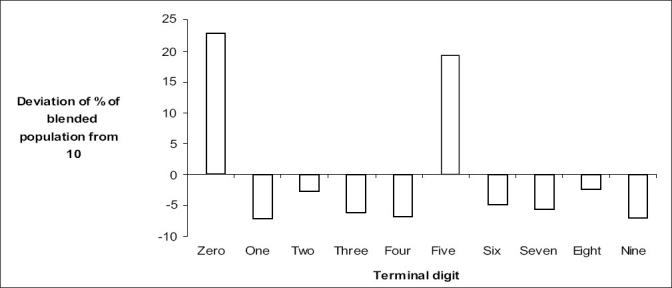
Myers index by terminal digit

Figure 1 describes the deviations of the percentage of the blended population from 10 along each of the terminal digits. The most preferred terminal digits while reporting age were ‘0’ and ‘5’ and most least mentioned were ‘1’, ‘9’, and ‘4’.

The total population aged up to 74 years was 4197. Table 2 describes the calculation of the age ratios for males and females in this population. The age ratio for males was 13.06 and for females was 16.35. The AAI for the population studied was 14.71. Maximum positive deviations in males was observed in the age group of 65–69 years (26%) while in females it was at 60–64 years (44%). The maximum negative deviations were noted in the 55–59 years age group (33%) in males and in the 55–59 year age group (28%) in females.

**Table 2:** Age accuracy index for males and females

Age (years)	Analysis of age ratio					
	Population		Male		Female	
	Male	Female	Ratio	Deviation from 100	Ratio	Deviation from 100
	1	2	3	4	5	6
<5	161	151				
5–9	207	206	102.31	2.31	101.31	1.31
10–14	239	253	103.31	3.31	110.31	10.32
15–19	248	229	103.91	3.91	107.84	7.85
20–24	229	155	110.63	10.63	80.44	-19.55
25–29	144	194	85.21	-14.79	114.56	14.57
30–34	134	159	87.96	-12.04	87.36	-12.64
35–39	179	193	111.88	11.88	127.53	27.53
40–44	167	102	111.83	11.83	79.89	-20.10
45–49	102	88	84.30	-15.70	98.14	-1.86
50–54	94	79	116.05	16.05	105.80	5.80
55–59	47	57	66.82	-33.18	72.45	-27.54
60–64	70	100	107.69	7.69	143.54	43.54
65–69	78	52	126.49	26.49	80	-20.00
70–74	37	43				
Total (irrespective of sign)				169.80		212.61
Mean				13.06		16.35

**Figure 2:**
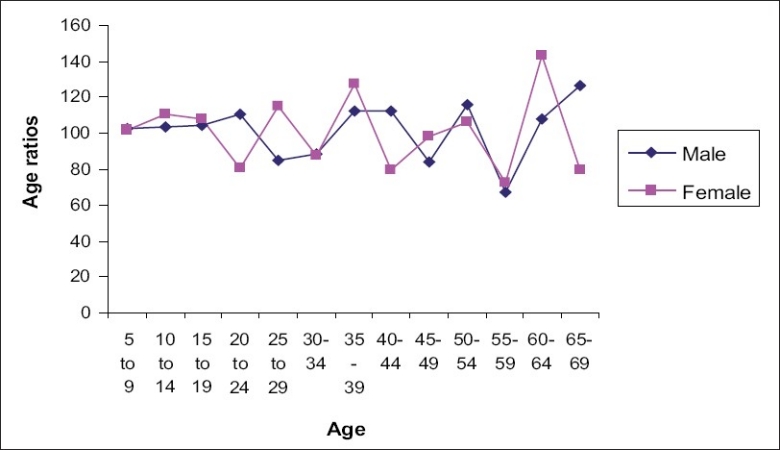
Age ratios by sex for five year age groups

Figure 2 describes the age ratios according to sex for the 5-year age group. The curve is not smooth but shows sharp jumps and clustering at certain ages indicating large differences between frequencies of populations in adjacent groups. A comparison of the curves for males and females indicates large differences between frequencies of males and females at certain ages. For example, in the age group of 20–24, the males show a positive deviation while in the case of females, a sharp dip is noted. A reverse phenomenon is seen in the age group of 25–29 years.

When the interviewers introduced themselves and requested the household members to provide information regarding age, one of the family members usually volunteered to give the information. If he/she was not sure about someone’s age, it was cross-checked with other members.

Some of the responses which indicate difficulties in data collection were as follows:

*Tumhich bagha ata majhe vay kiti asel te* (You only decide my age)

*Majhe vay andaje liha* (Write my approximate age)

*Mahit nahi* (I do not know my age)

*Majhe vay pastis te chalis asel* (My age must be around 35–40)

Some wanted to know the objective of collecting data.

*Kasha sathi? Ya mule kay fayda ahe?* (Why do you want to know my age? What benefit will I get?)

In such situations a few hints were given by the interviewers such as age at marriage, duration of marriage, duration after marriage when the first child was born, and in the case of children, age was ascertained depending on the class of school in which the child was studying. Some of the interviewers stated that they made only rough estimates of the age in such cases.

## Discussion

The methodology for data collection used in this study was similar to the method used in nearly all communitybased studies. Considering the three indices studied, the quality of age data collected in the survey can be inferred to be of very poor quality. There was age heaping at ages with terminal digits ‘0’ and ‘5’, indicating a preference in reporting such ages. The accuracy of age data should be assessed using various indices in studies in which age is an important variable.

Whipple’s index is considered to be a fair measure of general reliability of age distribution. The ages of early childhood and old age are excluded from the formula because they are more frequently influenced by other types of errors and issues than digit preference. Whipple’s index of more than 175 indicates that age distribution is very rough with age heaping at ages with terminal digits ‘0’ and ‘5’.(4) Myers’ blended index for the study group indicates that a minimum of 40% of the population reported ages with an incorrect final digit. No cut-off values for AAI have been described. Among the 42 countries for which AAI was calculated in a study, a majority had AAI less than 10 except for two countries, namely, UAE (United Arab Emirates) and Russia in which AAI was more than 14, and were categorized as high AAI.(5) Lower the value of AAI, the more accurate the age data. In this study, AAI brings out irregularities other than age heaping at terminal digits ‘0’ and ‘5’. These include differences in the frequency of population in adjacent age groups and in males and females in the age groups studied.

The approximation of age awareness manifests itself in the phenomenon of age heaping in self-reported or proxy age data. Individuals lacking knowledge of their age rarely state this openly, but choose instead a figure they think plausible. They do not choose randomly but have a systematic tendency to prefer attractive numbers such as those ending in ‘0’ and ‘5’ or even numbers or in some societies, numbers with other specific terminal digits. Age heaping indicates ignorance of one’s own age or a tendency to round ages. Age awareness is quite low and many have only a vague idea about their age. In cases where age is reported by proxy respondents, the response is more likely to be an approximation or a guess.(6) The role of the respondents and interviewers leading to age heaping has not been differentiated in this study.

Age heaping has been noted in studies which have analyzed age data in census, Demographic and Health Survey (DHS), and National Family Health Survey (NFHS) in India.(1,7,8) A number of determinants of age heaping such as literacy, household size, degree of interaction with administration, use of calendars, astrology, etc. have been studied.(8,9)A strong and statistically significant association has been found between age heaping and illiteracy and age heaping has been used as an indicator of human capital.(10)

The impact of such misreporting can lead to misclassification bias and wrong assessment of demographic rates and interfere with planning effective interventions. The official records such as birth certificates and school certificates can be a valid source of information regarding age but such records may not be available in many households. NFHS-3 survey in Maharashtra has reported that among children under 5 years of age, 80% births were registered; but in 35% children, births were registered but their birth certificates were not available.(11)

Other methods of data collection which ensure the accuracy of age data need to be evolved. In a study, a local time path calendar was used in which the interviewer took the respondent back in time using the local calendar and the memory of respondents was triggered by relating events to Indian festivals and other landmarks in the lives of people, enabling them to reply in their own time perspective.(12) The findings indicated significantly less heaping in the durations of postpartum amenorrhea, breastfeeding, postpartum abstinence, and contraceptive use.

The quality of age data is important because age sex distribution is not only an invariable part of a survey report but the bias introduced in studies can lead to wrong inferences. Innovative methods in data collection along with measuring and minimizing errors using statistical techniques should be used to ensure accuracy of age data.
